# Identification of Policy Priorities to Address the Burden of Smokeless Tobacco in Pakistan: A Multimethod Analysis

**DOI:** 10.1093/ntr/ntz163

**Published:** 2019-08-29

**Authors:** Kamran Siddiqi, Ziauddin Islam, Zohaib Khan, Faraz Siddiqui, Masuma Mishu, Omara Dogar, Vandana Shah, Javaid Khan, Subhash Pokhrel, Romaina Iqbal, Linda Bauld, Aziz Sheikh, Jean Grugel, ASTRA

**Affiliations:** 1 Department of Health Sciences, University of York, York YO10 5DD, UK; 2 Tobacco Control Cell, Ministry of National Health Services, Regulation and Coordination, Islamabad, Pakistan; 3 Office of Research Innovation and Commercialization, Khyber Medical University, Peshawar, Pakistan; 4 Campaign for Tobacco Free Kids, Washington, DC 20005; 5 Department of Medicine, Aga Khan University, Karachi, Pakistan; 6 Department of Clinical Sciences, Brunel University London, Uxbridge, Middlesex UB8 3PH, UK; 7 Department of Community Health Sciences, Aga Khan University, Karachi, Pakistan; 8 Usher Institute of Population Health Sciences and Informatics, University of Edinburgh, Edinburgh EH8 9DX, UK; 9 Department of Politics, University of York, York YO10 5DD, UK

## Abstract

**Introduction:**

We assessed the magnitude of smokeless tobacco (ST) use in Pakistan and identified policy gaps to help ascertain short-, medium-, and long-term priorities. We then elicited stakeholders’ views as to which of these identified priorities are most important.

**Methods:**

In a multimethod study, we: analyzed Global Tobacco Surveillance System data sets to estimate ST consumption and disease burden; conducted a documentary review to identify gaps in policies to control ST in comparison with smoking; elicited stakeholders’ views in an interactive workshop to identify a set of policy options available to address ST burden in Pakistan; and ranked policy priorities using a postevent survey.

**Results:**

Among all tobacco users in Pakistan (*n* = 24 million), one-third of men and two-thirds of women consume ST. In 2017, its use led to an estimated 18 711 deaths due to cancer and ischemic heart disease. Compared to smoking, policies to control ST lag behind significantly. Priority areas for ST policies included: banning ST sale to and by minors, advocacy campaigns, introduction of licensing, levying taxes on ST, and standardizing ST packaging. A clear commitment to close cooperation between state actors and stakeholder groups is needed to create a climate of support and information for effective policy making.

**Conclusions:**

Smokeless tobacco control in Pakistan should focus on four key policy instruments: legislation, education, fiscal policies, and quit support. More research into the effectiveness of such policies is also needed.

**Implications:**

A number of opportunities to improve ST regulation in Pakistan were identified. Among these, immediate priorities include banning ST sale to and by minors, mobilizing advocacy campaign, introduction of licensing through the 1958 Tobacco Vendors Act, levying taxes on ST, and standardizing ST packaging.

## Introduction

Smokeless tobacco (ST) is consumed by approximately 356 million people (one-fourth of all tobacco users) in 140 countries.^1^ In 29 of these countries, more than 10% of the population uses ST regularly.^2^ Regulated poorly,^2^ ST products vary in their composition and health risks. Most of these products, particularly in low- and middle-income countries (LMICs),^3^ contain high levels of nicotine and nitrosamines, leading to dependence and a high incidence of head and neck cancers, respectively.^4^ ST products are also associated with cardiovascular deaths^5^ and, if consumed during pregnancy, with poor birth outcomes.^6^ The burden of disease due to ST use is particularly high in Asia and Africa,^3,7^ where the poorest sectors of society bear the greatest burden.^1^

Despite being signatories to the World Health Organization (WHO) Framework Convention for Tobacco Control (FCTC), there is substantial disparity in policy development and implementation between smoking and ST products.^2^ As a consequence, progress to curb ST consumption is falling behind that for smoking.^2^ In Pakistan—a high tobacco burden country—ST products are popular, cheap, and easily available.^8^ Unlike cigarettes, ST consumption is also common and socially acceptable among women and young people.^9^ Recently, several academics, civil society organizations, and statutory bodies from Pakistan have joined a new initiative called Addressing ST and building Research capacity in south Asia (ASTRA).^10^ On behalf of ASTRA, we conducted a multimethod analysis to identify policy challenges, opportunities, and priorities to improve its regulation.

## Methods

We: (1) analyzed secondary data to describe ST use prevalence and estimate its disease burden; (2) reviewed documents to compare policies for controlling ST with those for smoking; (3) convened an interactive consultation to elicit stakeholders’ views to identify a set of policy options available to address ST; and (4) conducted a postevent survey to determine the most important policy priorities.

### Secondary Data Analysis

We analyzed Global Adult Tobacco Survey^11^ (GATS) 2014 and Global Youth Tobacco Survey^12^ (GYTS) 2013 to compare distributions of ST use with smoking. The disease burden attributable to ST use was estimated as a proportion of the burden reported in the 2017 Global Burden of Disease study.^13^ For this, we used comparative risk assessment method—also used previously to estimate global burden of disease due to ST.^3^ Exposure to ST was extrapolated from GATS,^11^ and disease-specific risk estimates for cancers and cardiovascular diseases were obtained from previously published meta-analyses.^3,5^

### Documentary Review

We undertook a comparative documentary review to highlight differences in policies between smoking and ST. We reviewed all tobacco-related legislative documents and Statutory Regulatory Orders (SROs) issued in the last 20 years by the then Ministry of Health and Ministry of National Health Services, Regulations and Coordination Pakistan (NHSRC).^14^ We also reviewed summaries of the legislation available on tobacco control laws web site.^15^ Moreover, we analyzed GATS^11^ data to assess the extent to which advice and support is offered to ST users in comparison with smokers.

### Priority Setting Workshop

We shared the findings of the above two studies at a 2-day stakeholders’ workshop. We invited a total of 28 policy makers, academics, and advocates at this event and organized discussions around five themes:^16^ fiscal policies, product regulation, education and awareness, cessation support, and knowledge gaps. Following the initial brainstorming and discussion at the event, a consensus development approach was undertaken to identify and agree on key priorities to address ST use. After the event, the key priorities were shared with all 28 stakeholders for feedback.

### Postevent Survey

Postworkshop, the stakeholders used an online survey to rank these policies on a five-point scale in terms of their priority level. Stakeholder’s responses were pooled to generate mean scores for the policy options and ranked accordingly.

## Results

### Prevalence of ST Use and Associated Disease Burden

Over 9.6 million adults (7.7% overall, 11.4% males, 3.7% females) in Pakistan consume ST regularly; this constitutes approximately one-third (35.6%) of all male tobacco users and up to two-thirds (62.8%) of all females’ tobacco users. Among adolescents aged 13–15 years, up to 54.0% of tobacco users consume ST, with estimates of current use being 6.4% and 3.7% in males and females, respectively. *Naswar* is by far the most commonly used ST product, followed by tobacco containing *paan* and *gutkha*.

The overall disease burden attributable to ST was based on the risk of mouth (relative risk [RR] 5.2), pharyngeal (RR 2.6) and esophageal (RR 2.6) cancers, and ischemic heart disease (RR 1.57).^3^ We estimated that, in 2017, approximately 18 711 deaths and 200 638 disability-adjusted life years (DALYs) in Pakistan were attributed to ST users. A third of this disease burden was estimated to be due to cancers and two-thirds due to ischemic heart disease.

### Policy Gaps in Relation to ST

**Figure 1.**
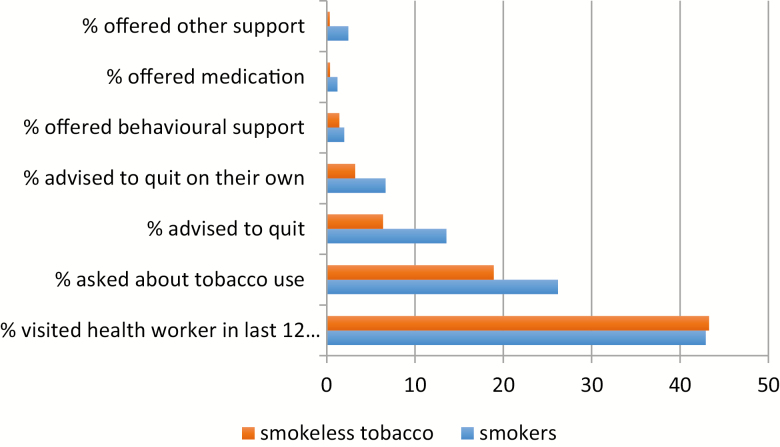
A comparison between smokers and smokeless tobacco users in the way they are advised by health professionals in the last 12 months.

Table 1 lists the tobacco control laws in Pakistan, identifying the differences between smoking and ST. Other than restricting general advertisement and sale within the vicinity of educational institutions, all other laws have not been applied to ST, indicating a wide disparity in the way the two types of tobacco use are dealt with. Further analysis of GATS results (Figure 1) found that health professionals are less likely to inquire about individuals’ ST use as compared to cigarette use and also less likely to offer advice, behavioral support, or medication to help them quit (Figure 1). On the other hand, knowledge and awareness of health-related harms of tobacco use were lower among ST users (77.0%) than in smokers (86.0%).

**Table 1.** A comparative analysis of tobacco control laws in Pakistan for both smoking and smokeless tobacco

Tobacco control laws in Pakistan	Smoking	Smokeless tobacco
Requirements to have nicotine and tar contents on the label	No	No
Requirements to have excise stamp, affixing banderols on the pack	No	No
Prohibition on quantity, i.e., sale less than 20 per pack	Yes	No
Smoke-free laws	Yes	Not applicable
Warning labels on packs	Yes	No
Restriction on advertisement of tobacco	Yes	Yes
Promotion of samples to minors	Yes	No
Prohibition of sale of tobacco to minors and by minors	Yes	No
Prohibition of storage, sale, and distribution of tobacco in the immediate vicinity of educational institutions	Yes	Yes

### Barriers and Opportunities

In the stakeholders’ workshop (attended by 19/28 invitees), there was acknowledgment of the gaps in ST regulations and the need to reduce ST-related burden. Participants recognized that ST is manufactured primarily in the informal sector in Pakistan, which made it challenging to tax and regulate. However, unlike cigarettes, this also made any direct industry interference less likely in response to any policy action(s). Other barriers included lack of awareness of the ST-related health risks among policy makers and legislators, variations in packaging of ST products, little understanding of its supply chain, and an absence of evidence on cessation strategies. It was also recognized that, over the long term, policies to reduce ST use should include encouraging alternative livelihoods for producers.

### Policy Priorities

The following priorities were identified (and ranked by 20/28):

1. Fiscal policies: Ninety percent considered the enactment of the 1958 Tobacco Vendor Act that mandates licensing for all tobacco sellers as high priority. Seventy-five percent considered levying taxes at a minimum of 70% of the retail price, implementing tax track and trace and earmarking tobacco tax for providing alternative livelihoods as high priorities.
2. Cessation services: There was a significant majority of participants who considered the formulation of national guidelines for treating tobacco dependence (80%), integrating tobacco dependence treatment within existing health systems (75%), and incorporating it within health professionals’ curriculum (70%) should receive high priority. A comprehensive service to treat tobacco dependence was considered a long-term goal.
3. Education and awareness: A comprehensive media campaign to raise public awareness about ST (90%) and building an antitobacco advocacy coalition of legislators, health professional, journalists, cancer charities, and patient support groups should be the highest priority (90%), followed by antitobacco messages within educational institutions and curricula (80%).
4. Product regulation: Everyone agreed that a ban on ST product sale to and by minors should be introduced immediately. Seventy percent considered standardizing ST packaging and labeling including mandating pictorial warnings and formalizing industry by introducing licensing should be a high priority. It was agreed that manufacturers requiring disclosure of their ingredients and regular inspections and laboratory testing could be introduced at a later stage.
5. Research priorities: These included an estimation of the economic and disease burden of ST use and the return on investment in addressing it. Others included documenting all ST products and their composition available in Pakistan, their market share, and points in the supply chain where taxes can be levied.

## Discussion

ST products are commonly used in Pakistan and impact different segments of population, including women and people of low socioeconomic status. Products consumed are known to contain high levels of nicotine and nitrosamines, which are linked to wide-ranging harms including specific types of cancers (head and neck), cardiovascular diseases, and poor perinatal outcomes. The profile of ST products in Pakistan is in contrast with those in Nordic countries, where available ST products have low nitrosamine levels and pose fewer health risks.^17^ Moreover, unlike smoking, ST has received little attention so far. This is the first priority setting exercise that we know of to provide a list of policy actions to address ST use in Pakistan.

Among all 140 countries where ST is consumed, Pakistan is among the top three countries with the highest ST-related disease burden, superseded only by India and Bangladesh.^3^ A recent global policy review^2^ highlighted that a majority of the 140 countries have not yet put comprehensive tobacco control measures covering ST use in place. Only a handful of countries levy taxes up to 70% of their retail price, inspect their contents, ban advertising, or warn their populations of ST-related harms. That said, Pakistan could learn lesson from India where some policy action has happened to curb ST use, including effective advocacy led by civil society in India, leading to the introduction of strong pictorial warnings, use of Food Safety and Standards Act, 2006, to ban gutkha sale, levying tax on ST manufacturing units, and prohibiting ST use as an ingredient within tooth cleaning products.^18^

### Limitations

Our disease burden figures are conservative and are derived from risk estimates based on ST use in Pakistan and other countries.^3,5^ The stakeholders’ views included in this study come from a nonrandom sample of workshop and postevent survey participants. They include a cross section of wider stakeholder group (health advocates, health care providers, and researchers) within Pakistan’s health sector. Policy makers’ views may be underrepresented; this is a gap ASTRA will address in the near future. However, the policy elicitation process was facilitated by international experts and supported by health professionals from Pakistan who are knowledgeable and experienced in the field. Despite its limitations, it constitutes the first initiative to consolidate and coordinate research and advocacy expertise in ST use in Pakistan.

### Implications

The policy priorities identified in our analysis are similar to global priorities for ST,^2^ for example, taxation, standardization of packaging, educational campaigns, training health professionals in treating ST dependence, preventing the sale by and to minors, and monitoring the health and economic impact. Were even some of these policies to be adopted, it is likely that the impact of ST on health outcomes would be reduced, though research will, of course, be needed to establish which interventions produce the most significant health impact. With this in mind, the above analysis was also useful in orientating ASTRA’s future research direction in line with the knowledge gaps identified by the stakeholders and in building capacity and generating demand for further research—one of ASTRA’s objectives. For all affected communities, it is hoped that this exercise will generate momentum for a multisectorial effort to address ST as a public health issue.

## Funding

This study is funded by the National Institute for Health Research, UK, and also by the Global Challenges Research Fund, UK.

## Declaration of Interests

None declared.

## Author Contributions

KS, FS, ZK, OD, JG, and ZI conceptualized the study. KS, ZK, FS, OD, and ZI collected and analyzed the data. All authors interpreted the results and contributed in writing, revising, and editing different drafts of the manuscript.
